# Editorial: Molecular modeling in drug repurposing

**DOI:** 10.3389/fmolb.2026.1857864

**Published:** 2026-05-06

**Authors:** Hugo A. L. Filipe, Rebeca Garcia-Fandiño, Ángel Piñeiro, Carlos Fernandez-Lozano

**Affiliations:** 1 BRIDGES - Biotechnology Research, Innovation, and Design of Health Products, Polytechnic of Guarda, Guarda, Portugal; 2 Department of Organic Chemistry, Center for Research in Biological Chemistry and Molecular Materials, Santiago de Compostela University, CIQUS, Santiago de Compostela, Spain; 3 MD.USE Innovations SL, Edificio Emprendia, Santiago de Compostela, Spain; 4 Department of Applied Physics, University of Santiago de Compostela, Santiago de Compostela, Spain; 5 Department of Computer Science and Information Technologies, Machine Learning in Life Sciences Laboratory, Universidade da Coruña (CITIC), A Coruña, Spain

**Keywords:** artificial intelligence, disease targets, drug repurposing, machine learning methods, molecular docking simulations, molecular dynamics simulations, protein-ligand interaction

Drug repurposing seeks new therapeutic uses for compounds that already have established safety and pharmacokinetic profiles. Because *de novo* drug development remains slow, expensive, and prone to failure, repurposing has attracted growing interest from both academia and industry. Several European initiatives, including REPO4EU, REMEDi4ALL, and RePo-SUDOE, reflect this momentum and aim to build collaborative frameworks that accelerate the drug repurposing process. Molecular modeling techniques, particularly molecular docking and molecular dynamics (MD) simulations, play a central role in these efforts by providing atomic-level descriptions of how drugs interact with their targets. More recently, artificial intelligence (AI) methods, and in particular machine learning (ML), have been coupled with physics-based simulations to improve predictive accuracy and scale up virtual screening campaigns.

We organized this Research Topic to bring together work that uses molecular modeling, broadly defined, to advance drug repurposing. The Research Topic includes five articles covering pharmacophore design, transporter biology, network pharmacology, transcriptomics-guided docking, and RNA-protein targeting. Together, they reflect how diverse the computational toolkit for repurposing has become.

Elsaka et al. review pharmacophore modeling, from its origins as a theoretical concept to its current use as a routine virtual screening tool. Their discussion of dynamic pharmacophore models (dynophores) derived from MD trajectories is particularly relevant, as these models account for binding-site flexibility that static approaches miss. The review also covers recent applications of AI to feature extraction and hit-rate improvement, illustrated with case studies on efflux pumps, topoisomerase II-alpha, and LEDGF/p75-integrase inhibitors.

In a different therapeutic context, Kaijage and Kraszewski focus on sodium-glucose co-transporters (SGLTs) and the challenge of achieving selective SGLT1 inhibition. They bring together cryo-EM structures, AlphaFold2 predictions, and free energy calculations from MD simulations to propose a roadmap for designing selective inhibitors. The review highlights how structural data from different sources can be combined computationally to guide rational drug design for metabolic and cardiovascular targets.

Hu et al. take a data-driven approach to intestinal ischemia-reperfusion injury, using WGCNA and machine learning to identify five mitochondrial-related hub genes (Pdk4, Yrdc, Bcl2l11, Bcl2a1d, and Pmaip1). Molecular docking serves here as a validation step rather than the primary discovery method, linking the transcriptomic findings to potential therapeutic compounds, specifically securinine and ABT-737. This work illustrates how docking can complement omics-based pipelines even when it is not the driving methodology.

Prakash addresses brain cancers, a setting where tumor heterogeneity makes drug selection particularly difficult. The study builds molecular profiles from signaling pathway components (EGFR, BRAF, PDGFRA, TP53, CDKs) and screens 2,809 FDA-approved drugs through two purpose-built tools: “in-mac” for profiling and “ReBrain” as a network-based database. The framework reports 70%–95% accuracy and identifies mefloquine, clofibric acid, and armillarisin A as priority candidates, showing that network pharmacology can handle the complexity of heterogeneous tumors.

Finally, Smith et al. push the boundaries of what counts as a druggable target. Their perspective proposes ribonucleoprotein (RNP) interfaces in viral 5′ untranslated regions as sites for small-molecule intervention, using Enterovirus A-71 as a proof of concept. The workflow combines structural prediction, ensemble-based MD simulations, virtual screening, and biophysical validation. Targeting RNA-protein interaction surfaces for repurposing remains largely unexplored, and this contribution lays out a concrete strategy to address it.

Taken together, these articles point to a few recurring observations that we believe are worth highlighting. Modern repurposing campaigns rarely rely on a single computational method. Instead, the most convincing results come from combining docking, MD, network analysis, or ML in pipelines where each technique compensates for the limitations of the others. At the same time, the structural and dynamic characterization of drug-target interactions remains the foundation that gives computational predictions their mechanistic grounding, whether the target is a protein, a transporter, or an RNA-protein complex. Equally important, none of these computational strategies can stand alone without experimental validation to confirm that *in silico* predictions translate to measurable biological effects.

We believe this Research Topic shows that molecular modeling, in combination with data-driven and AI-based approaches, is now a practical and versatile component of the drug repurposing toolkit. The five contributions span therapeutic areas as different as viral infections, brain cancers, metabolic disease, and ischemia-reperfusion injury, yet they share a common reliance on computational methods to generate and prioritize hypotheses. We hope this Research Topic encourages further work connecting simulation, data science, and experimental validation in the service of finding new uses for existing drugs.

